# Physiological and Cognitive Functions Following a Discrete Session of Competitive Esports Gaming

**DOI:** 10.3389/fpsyg.2020.01030

**Published:** 2020-05-29

**Authors:** Amber Sousa, Sophia L. Ahmad, Tamzid Hassan, Kyle Yuen, Peter Douris, Hallie Zwibel, Joanne DiFrancisco-Donoghue

**Affiliations:** ^1^New York Institute of Technology (NYIT) College of Osteopathic Medicine, Old Westbury, NY, United States; ^2^NYIT Center for eSports Medicine, Old Westbury, NY, United States; ^3^OMS II, NYIT College of Osteopathic Medicine, Old Westbury, NY, United States; ^4^Department of Physical Therapy, New York Institute of Technology, Old Westbury, NY, United States

**Keywords:** esports, competitive gaming, executive functions, cognition, health

## Abstract

Competitive organized electronic video gaming, termed “esports,” has become an international industry. The physiological and cognitive health results of prolonged esport practice and competition have not been adequately studied. The current study examined physiological and cognitive changes after a session of esport gameplay for two types of games, first-person shooter and multiplayer online battle arena games. Increases in systolic blood pressure, increases in speed, and decreases in accuracy and inhibitory processes were found for esport gamers overall. For peak heart rate change, first-person shooter games elicited a larger change than did multiplayer online battle arena games. These results have implications for the management of esport player cognitive and physical health as well as for the optimization of performance in competitive esport tournaments.

## Introduction

The term “electronic sports” or “esports” refers to organized electronic and video game competitions that have recently exploded in popularity and are now a global multimillion-dollar industry (Willingham, 2018). Many colleges and universities already have esport varsity teams, and some even offer scholarships akin to traditional sports scholarships (Morrison, 2018). Although requiring less contact and physical activity than most sports, esports do share some commonalities with tradition athletic sports. For example, tournaments involve players either as individuals or teams facing opponents in games that are divided into rounds and matches. Players often train for hours each day, are coached professionally, and can receive sponsorships from companies (Jacobs, 2015). Many times, these players play for hours in practice and competition without any mental or physical break.

Esport games encompass a wide breadth of genres that are thought to require a unique skill set to play competitively (Norman, 2011). For example, *League of Legends* is a multiplayer online battle arena (MOBA) game. Teams are formed and tasked with the mission of destroying the opposing team’s base (core) while also defeating the opposing team members. In this game, characters are developed, requiring teamwork and strategy (Alloza et al., 2018). In contrast, *Overwatch* is a first-person shooter (FPS) game. FPS games put the player in the first-person perspective of their character and task them with various missions, such as defending or attacking an objective or protecting a fort. FPS games are different from MOBA games in that they are usually more fast-paced (Alloza et al., 2018). FPS games are meant to simulate the player’s visual field during gameplay occurring in real time. Therefore, faster decision making, strategizing, and reaction times are likely required to provide the best outcome. MOBA games, in contrast, place the player in the middle of the screen along with the surrounding map, allowing the player to be able to interact and anticipate moves from the opposing team, which may require a larger degree of strategizing. However, given that there are differences even between games within each genre, these assumptions are likely not universal truths, and little research informs the issue.

Few studies have examined the physical and physiological effects of esports. Tournament conditions may be similar to traditional sport competitions in that they both produce stress for the player. However, the type of stress is likely quite different; esports do not involve the level of physical exertion required for many other sports but may elicit a psychological state of stress or tension that results in autonomic nervous system alteration. Esports also seem to involve some degree of physical injury. One study listed the common complaints of esport players to include joint pain, headaches, sleep problems, and vision problems (Benchebra et al., 2019), and in another study of collegiate esport players, digital eye strain was the most common reported ailment (DiFrancisco-Donoghue et al., 2019).

Executive functions, the cognitive functions that govern our behavior, decision-making abilities, organization, planning and goal setting, time management, and self-regulation, are likely utilized during gameplay. An esport player simultaneously performs complex actions while analyzing various amounts of stimuli in order to create a fluent and coordinated action, while trying to minimize the amount of erroneous choices that may be detrimental to their desired goals. Some studies have found that people who play video games may exhibit faster response times but reduced accuracy on some executive functioning measures (Kowal et al., 2018). Similarly, a study of FPS and MOBA game types found that gamers who played FPS presented faster reaction times but a lower control over inhibition than did gamers who favored MOBAs (Deleuze et al., 2017).

The primary purpose of this study was to examine potential changes in executive function and physiological markers following a discrete session of competitive gaming. We anticipated physiological changes associated with increased sympathetic nervous system stress. We were also interested in potential changes in executive functioning that may result from prolonged esport gameplay.

## Methods

### Design

This study was a prospective observational cohort study, which allowed investigation of the temporal sequences between exposure and outcome and investigation of the effects of one exposure on multiple outcomes. The outcome measures including both cognitive and physiological measures were examined before and after a session of esport gaming across two types of esport games (FPS and MOBA).

### Setting

Data were collected at the CyBears Arena at New York Institute of Technology (NYIT), Old Westbury, United States. The room consists of gaming computers, monitors, headsets, specialized gaming keyboards, and mouses, set up around the perimeter of the room. The data were only collected from tournament settings, including competing against another school for a prize or playing using ranked settings online against strangers to gain or lose ranking. Playing ranked online utilizes the same format and rules as is in competition against other schools.

All members of the NYIT CyBears reported to the room during competition times to have their data collected before and after a discrete gaming session. The opposing team was not physically present.

### Sample

This study was approved by the NYIT Internal Review Board, and all subjects signed written consent after the study was explained to them and they were given time to ask questions. Subjects were recruited from the student population at NYIT, Old Westbury, NY, United States. Esport players in the age group of 18–30 were recruited through Discord, the communication application used by members of the CyBears esport team. Inclusion criteria included women or men age 18 to 30 years who were members of the NYIT esport team. Exclusion criteria included any player that did not participate in competition for the esport team or who had any current injury that would affect typical gameplay.

### Data Collection

Data were collected immediately before and after a gaming session. Prior to a match, measurements included blood pressure (BP), heart rate (HR), respiratory rate (RR), visual acuity, and a measure of psychomotor speed [finger-tapping test (FTT)]. In addition, participants were administered a series of online executive function tests. Subjects were then fitted into a Hexoskin^®^ shirt, which monitored their HR throughout gameplay.

At the completion of the match (approximately 2.5 h depending on the natural length of type of game played), the subjects repeated the online cognitive function tests. This was done to ensure that members did not break eye contact from the screen immediately after gameplay, followed by measurement of visual acuity. After that, participants had their vital signs and FTTs completed one additional time.

### Outcome Measurements

#### Visual Acuity

Visual acuity was measured using a standard eye chart (Snellen chart). First, subjects stood 6 m away from the chart and were asked to occlude one eye while viewing the chart with the other eye. Subjects then read the letters on each line, starting from the top of the chart and reading left to right, and continued until they failed to correctly identify a letter on a line (Marsden et al., 2014). Because digitally strained eyes begin to recover when the eyes are not looking at a screen, the eye exam was given as soon as the subjects finished practicing.

#### Blood Pressure

Blood pressure was taken manually after sitting quietly following 5 min for initial measure. For the post-gameplay measure, BP was taken upon completion of cognitive measures along with HR and RR.

#### Mental Flexibility

The Trail Making Test (TMT) is a neuropsychological instrument that evaluates speeded visual search (Corrigan and Hinkeldey, 1987). A computerized version of the TMT, whereby subjected used a mouse to connect numbered and lettered circles in chronological and alphabetical orders, was administered through Inquisit Lab, produced by Millisecond. The test itself took no longer than 3 min and was completed prior to and after the subjects’ gaming sessions.

#### Inhibition

##### Color-word stroop with keyboard

The Stroop Color and Word Test is a measure of speeded inhibitory control and set switching (Stroop, 1935; Scarpina and Tagini, 2017). We utilized a computerized version of the Color-Word Stroop by Inquisit Lab, developed by Millisecond. The software measures the time to reaction in milliseconds as well as accuracy of responses. The test took 3–5 min to complete. This measure was administered before and after gaming sessions.

#### Psychomotor Speed

The FTT is a quantitative tool used as a measure of psychomotor speed in many standard neuropsychological evaluations (Halstead, 1947). It measures the motor speed of the index finger on both the dominant and non-dominant hands as a proxy evaluation of cortical motor area and efferent motor pathway integrity and motor functioning. Upon administering the test, the subject’s hand is placed on a wooden board with a pushable lever and counter while the administrator is tracking the taps. The test was completed within 5–10 min. This measure was done prior to and after the esport session was completed.

#### Physiological Variables

##### Hexoskin

During gameplay, subjects wore a Hexoskin Smart Shirt^®^ (5800 Denis St. Montreal, Quebec H2S-3L5), which is a garment with electrodes that can measure and calculate a number of physiological variables, including HR, HR changes, RR, steps taken, and sleep. Hexoskin’s validity in measuring these variables has been established (Cherif et al., 2018). The Hexoskin is worn on bare skin, with conductance gel applied to the electrodes to obtain accurate measurements. The shirt was worn for the entire gaming session. Following each session, the Hexoskin was connected to a computer, and the data were uploaded to the associated program for analysis.

### Statistical Analysis

IBM SPSS Statistics Version 25 was used to complete the statistical analysis. For each outcome variable, a repeated-measures general linear model was run with two levels. The within-subjects factor was the outcome variable, and a between-subjects factor of “type of game” was also used. Normality of distribution of data was ensured for each of the outcome variables. Significance level of *p* = 0.05 was used to determine statistical significance.

## Results

### Demographics

Data from all 17 subjects were utilized for the analysis. Subjects were all of male gender, average age was 20 years (SD = 1.82 years), and all but one participant were right-handed. Demographic information is listed in Table 1. Subjects’ gaming sessions lasted on average 151 min (SD = 49 min).

**TABLE 1:** Demographic information and performance on outcome variables broken down by type of game players.

	FPS game players (*Overwatch*) *N* = 9	MOBA game players (*League of Legends*) *N* = 8	Total group *N* = 17
Age	19.4 (1.3)	20.8 (2.1)	20.1 (1.8)
Gender (% male)	100	100	100
Handedness (% right-handed)	88	100	94

*FPS, first-person shooter; MOBA, multiplayer online battle arena.*

### Physiological Outcome Measures

**FIGURE 1:**
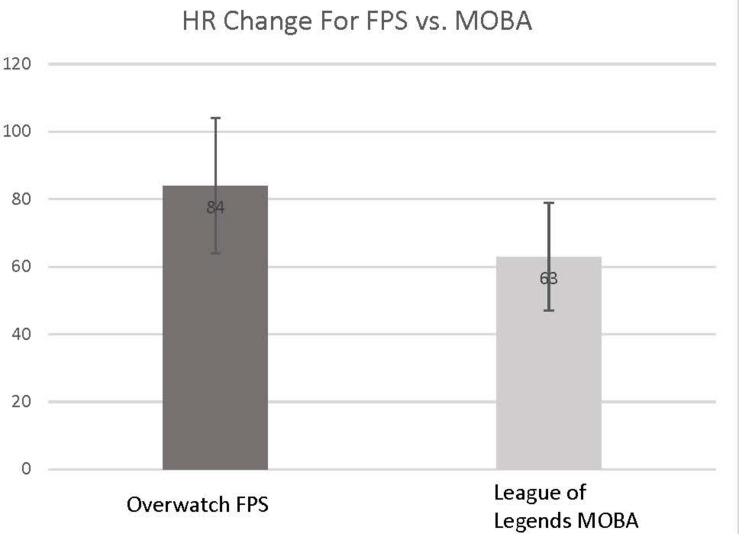
Maximum heart rate change observed for FPS vs. MOBA game type.

For physiological variables, we found that systolic BP significantly increased after game play session for the FPS game (*t* = -2.94, *p* = 0.019). For the MOBA game, systolic BP was decreased but not significantly (*t* = 1.44, NS). Respiration rate during gameplay increased at trend levels for the whole group (*F* = 4.24, *p* = 0.057). HR was not significantly different after gameplay session for either game type (*F* = 0.30, NS). Table 2 shows these results. However, the change from resting HR to peak HR during gameplay was significantly different for game types; for FPS game, HR increased significantly more during play than for the MOBA game (*t* = 2.22, *p* = 0.043). Figure 1 shows these results.

**TABLE 2:** Pregaming and postgaming comparison of physiological outcome variables with *F* statistic and *p*-values.

Outcome measure	Pregaming Mean (SD)	Postgaming Mean (SD)	Pre–post comparison (*F*)	Significance (*p*)	Interaction with game type (*F*)	Interaction with game type (*p*)
Systolic blood pressure	122 (10)	125 (12)	0.62	0.44	7.3	0.016*
Systolic blood pressure (*Overwatch*)	121 (10)	130 (8)	*t* = −2.94	0.019*		
Systolic blood pressure (*League of Legends*)	124 (10)	119 (13)	*t* = 1.44	0.12		
Heart rate	78 (8)	84 (14)	2.68	0.12	0.30	0.59
Respiration rate	15 (4)	17 (3)	4.24	0.057**	0.50	0.49
Vision	24 (6)	23 (5)	1.98	0.18	0.21	0.66

*Significant at the *p* = 0.05 level. **Non-significant but at the trend level.

### Neuropsychological Outcome Measures

Psychomotor speed as measured by the FTT was significantly faster after gameplay for the dominant hand (*F* = 11.68, *p* = 0.004) but was not significantly changed from pregaming to postgaming for the non-dominant hand (*F* = 0.00, *p* = 0.95). For executive functions, numerical sequencing speed (TMT A) was not significantly changed after gameplay (*F* = 0.19, *p* = 0.67). However, while performance on a measure of mental flexibility (TMT B) was significantly faster (*F* = 8.32, *p* = 0.011), the number of errors made increased, but only at a trend level of significance (*F* = 3.35, *p* = 0.087). On the Tower of London test, a measure of problem-solving ability, while accuracy was not significantly changed after gameplay (*F* = 0.12, *p* = 0.73), speed was significantly increased (*F* = 7.79, *p* = 0.014). Finally, on the Stroop test, a measure of impulsivity and response inhibition, while reaction time decreased and subjects were faster after gameplay (*F* = 5.89, *p* = 0.028), they made a significantly higher number of errors and were less accurate (*F* = 4.95, *p* = 0.042). Table 3 shows differences between pregaming and postgaming time points as well as interaction analyses when participants were grouped by type of game.

**TABLE 3:** Pregaming and postgaming comparisons of neuropsychological outcome variables with *F* statistic and *p*-values.

Outcome	Pregaming	Postgaming	Pre–post	Significance	Interaction with	Interaction with
measure	Mean (SD)	Mean (SD)	comparison (*F*)	(*p*)	game type (*F*)	game type (*p*)
Finger tapping (non-dominant hand)	47 (15)	46 (7)	0.00	0.95	0.68	0.42
Finger tapping (dominant hand)	48 (7)	52 (7)	11.68	0.004*	0.10	0.76
Trail Making Test Part A (speed in seconds)	25 (5)	24 (4)	0.19	0.67	1.37	0.26
Trail Making Test Part B (speed in seconds)	36 (12)	31 (10)	8.32	0.011*	0.84	0.36
Trail Making Test (total errors)	2 (1)	3 (2)	3.35	0.087**	0.05	0.82
Tower of London number correct	30 (6)	30 (5)	0.12	0.73	1.14	0.30
Tower of London (time)	8 (2)	7 (2)	7.79	0.014*	2.32	0.15
Stroop accuracy	0.95 (0.05)	0.91 (0.06)	4.95	0.042*	2.84	0.11
Stroop speed	0.8 (0.1)	0.7 (0.1)	5.89	0.028*	0.05	0.83

*Significant at the *p* = 0.05 level. **Non-significant but at the trend level.

## Discussion

Esports have rapidly become an ever-expanding industry with a large competitive arena. Many colleges and universities have incorporated formal esport teams into their offered varsity sports and scholarships. This has brought the health and well-being of esport athletes into the spotlight and the safety of long periods of competitive gaming into question. Yet to be made are recommendations for time-period parameters requiring rest of body or mental processes. This is important both from the perspective of the esport athletes’ health and to optimize playing ability in competitive tournaments.

In this study, we investigated the effects of esport gaming over a period of approximately 2.5 h for two types of games, FPS and MOBA. For physiological outcomes, our findings suggest that esport activity can increase sympathetic nervous system activation. Our findings that FPS gaming resulted in a larger change in low to peak HR and systolic BP increase when compared with the MOBA game suggests that FPS games elicit more of a sympathetic nervous system response. Other researchers have also found more aggressive or violent video games to elicit a cardiovascular stress response and a systolic BP increase (Siervo et al., 2013; Porter and Goolkasian, 2019). Still, others have found that gamers’ HR and electrodermal activity correlated with gamers’ subjective game-playing experience (Drachen et al., 2010). This may be a reflection of our FPS game, *Overwatch*, being more subjectively fast-paced than *League of Legends*. Our physiological findings may lead one to argue that esports can elicit an “aerobic” response, but this would in all likelihood be in error, akin to comparing an anxiety attack to health-promoting exercise. The main difference is that esports are likely eliciting physiological changes due to catecholamines, or stress hormones produced by the adrenal glands, given the low level of physical exertion. True aerobic exercise elicits these changes owing to physical exertion and oxygen demands of working muscles.

The cognitive effects of gaming have been lesser studied. One meta-analysis examined the effects of action video games, or games involving simulated physical challenges such as fighting or shooting games, on cognition and found that the cognitive areas of top-down attention and spatial processing were improved (Bediou et al., 2018). Another systematic review examining the neural bases of video-gaming found that video game players show enhanced attention functions for some types of attention, some visuospatial functions, and cognitive control, or the ability to manage tasks or information simultaneously (Palaus et al., 2017). We examined the short-term executive function effects of esport gaming over a session of gaming. Executive functions are the cognitive abilities that humans possess that allow them to manage behaviors, regulate emotional responses, and problem solve. A number of interesting findings were revealed. In general, we found for two of our three neuropsychological measures that contained both speed and accuracy components that although esport players exhibited faster response times after having competed, they were generally less accurate and exhibited more impulsive response styles. This was true for our measures of mental flexibility and impulsivity. However, for our measure of problem solving, although time was also improved, no decrement in performance was apparent. One could hypothesize that while gaming increases the need for problem solving and speed, there is little real repercussion for impulsivity, and therefore, behavior follows suit. Similarly to our findings, other researchers have also found that inhibitory control was decreased for players of FPS games (Deleuze et al., 2017).

Our findings may also be relevant to informing those interested in maximizing gaming performance in competitive esports. A functional MRI (fMRI) study showed that activation in areas of the brain implicated in executive control was associated with better gaming performance (Wang et al., 2018). Our findings of reduced executive functioning accuracy after hours-long gameplay may indicate the need for breaks in gaming in order to mitigate what might be a type of cognitive fatigue. Given that esport players of FPS games can elicit upward of 500 moves per minute, a loss in accuracy could be detrimental to gaming performance.

Limitations of the current study include a small sample size, as more participants would increase power for this study. Also, although we examined outcomes measures pregaming and postgaming, we cannot explain from our data how long the significant changes persist after gaming sessions. Some work has already examined the long-term effects of gaming, but more is needed (see Krarup and Krarup, 2020). Additionally, the length of time of esport engagement was not a tightly controlled variable, as we instructed esport players to complete matches for 3 h but did not specifically control the variable. This was in part done to not add stress of having to stop gaming while competing in a match, which conceivably could induce stress. Finally, we did not directly examine how executive function changes over esport gaming sessions might affect gaming performance. Future work may benefit from exploring the direct relationship between executive function and game performance.

In conclusion, this study demonstrates differences in executive function and physiological variables before and after a discrete session of esport gaming and across two types of esport games. Although 2.5 h of continuous gameplay demonstrated greater speed, it resulted in less accuracy and more impulsivity. Further research is needed in a larger cohort of competitive gamers to establish optimal duration of play before a decline in performance or risks to health occurs.

## Data Availability Statement

The datasets generated for this study are available on request to the corresponding author.

## Ethics Statement

The studies involving human participants were reviewed and approved by the NYIT Institutional Review Board. The patients/participants provided their written informed consent to participate in this study.

## Author Contributions

SA, TH, and KY worked to design the research study and to run participants, organize the data, and complete sections of the manuscript. AS and PD advised design regarding neuropsychological measures. JD-D and HZ advised design regarding physiological measures. AS, JD-D, and PD completed the statistical analysis.

## Conflict of Interest

The authors declare that the research was conducted in the absence of any commercial or financial relationships that could be construed as a potential conflict of interest.
